# The Airport, Air Quality and Asthma (AAA) Indoor Air Intervention Trial for Children with Asthma: Protocol for a Community Based Study in South King County, Washington State

**DOI:** 10.21203/rs.3.rs-7181465/v1

**Published:** 2025-09-22

**Authors:** Christine Loftus, Pamela Lim, Jan Capps, Jeffry Shirai, Maria Tchong-French, Elena Austin

**Affiliations:** University of Washington; University of Washington; Public Health - Seattle King County; University of Washington; University of Washington; University of Washington

**Keywords:** Asthma, community health workers, indoor air intervention, outdoor air pollution

## Abstract

**BACKGROUND:**

For children with asthma, exposure to indoor air pollution increases the risk of a serious asthma exacerbation, which can be life-threatening. Interventions aimed at improving indoor air quality, including use of a portable air cleaner with a high-efficiency particle air (HEPA) filter, may reduce this risk; however, the effectiveness, feasibility and acceptability of HEPA air cleaners varies, and more research in various settings is needed.

**METHODS:**

In collaboration with a community health worker (CHW) delivered asthma education program, we are conducting a randomized clinical trial to evaluate the effectiveness of HEPA air cleaners to improve indoor air quality and child asthma health in South King County of Washington State, a vulnerable community impacted by air pollution from airports and highway traffic. A key feature of the Airports, Air Quality and Asthma (AAA) design is extensive integration of CHWs, including CHWs recruited from community-based organizations, into multiple aspects of the trial protocol. We aim to recruit up to N=60 children with asthma, randomized into intervention and less effective filtration (control) groups in a 1:1 ratio, conduct baseline assessments of indoor air quality and airway health, and collect repeated assessments of air quality and airway health during a three-month intervention period as well as after the trial concludes. Primary effectiveness outcomes are concentration of indoor air pollution during the intervention period and two measures of child airway health: change in asthma control score pre- and post-intervention and incidence of asthma symptoms during the intervention period. Several secondary outcomes related to air quality and child health will be explored as well.

**DISCUSSION:**

To our knowledge, this is the first trial of indoor air filtration and pediatric asthma health in a community highly impacted by airport-related air pollution. The close collaboration with a CHW-delivered asthma program is also unique and important for future translation of results to future public health programming. Study findings will inform future approaches to integrate HEPA air cleaners into existing CHW asthma education programs in this and similar communities.

**TRIAL REGISTRATION:**

The AAA research study was retrospectively registered at clinicaltrials.gov (identifier: NCT07047430; registration approved July 1, 2025).

## INTRODUCTION

Asthma is one of the most common chronic diseases of childhood, with an estimated prevalence of 6.5% in U.S. children under 18 years old in 2021.^1^ The prevalence varies substantially across subpopulations and is generally higher for minoritized groups and underinsured families.^2^ For example, 12.5% of non-Hispanic Black children in the US have asthma, nearly twice the rate of other racial and ethnic groups.^1^ Children with poorly controlled asthma are more susceptible to episodic, acute exacerbations (i.e., asthma “attacks”). These exacerbations can be serious and even life-threatening, resulting in unscheduled clinic visits, emergency room (ER) admissions, or hospitalizations.^2^ In 2013 alone, pediatric asthma in the U.S. cost an estimated $5.92 billion in healthcare services.^3^ Poorly controlled asthma also has significant impacts on child and caregiver quality of life. School-aged children with poorly controlled asthma miss more school days than children without asthma, and their caregivers also miss more workdays than those of children without asthma.^4^

One well-established trigger of asthma exacerbations is exposure to outdoor and indoor air pollutants, which can cause short-term increases in airway inflammation and lead to acute bronchospasm.^5–10^ Exposure to traffic-related air pollution (TRAP), emitted by vehicles and aircraft with combustion engines, is especially widespread. TRAP consists of complex mixtures of nitrogen dioxide, carbon monoxide, volatile organic compounds, particulate matter (PM) of various sizes and chemical composition, and other components.^11^ Particulate matter with an aerodynamic diameter of 2.5 microns or smaller, PM_2.5_, has been extensively researched in studies of air pollution health effects, including pediatric airway health.^12,13^ Previous work has attributed 18–42% of asthma cases to TRAP exposure, with urban areas experiencing twice the burden of rural locations.^14^ TRAP mixtures near airports are elevated in ultrafine particulate matter (UFP), particles of 0.1 microns or smaller, a pollutant of emerging concern for child health.^15^ UFPs are relatively understudied compared to larger particulates due to challenges in monitoring and predicting UFPs, though recent evidence suggests that UFPs can penetrate deep into respiratory tracts due to smaller particulate sizes, increasing risk of exacerbations for individuals with asthma.^16^

Children spend a significant amount of time indoors, and exposure to indoor air pollution is another significant risk factor for pediatric asthma morbidity.^17,18^ Indoor air pollution results from the infiltration of outdoor pollutants to indoor environments, as well as from indoor sources, including cooking, heating, smoking or vaping, candles or incense, use of cleaning products, pesticide applications, pets, pests, carpets and bedding. Notably, the concentrations of air contaminants indoors can exceed those in the ambient outdoor air in some conditions.^19–23^ Sociodemographic disparities in indoor air pollution exposures are well-documented, attributable to inadequate ventilation; presence of mold, mildew or pests; and other factors.^24^

More interventions supporting parents and children in reducing air pollution exposure are needed, especially those feasible to implement in low-resource communities. One such intervention is home-based asthma education programs administered by community health workers (CHWs), frontline public health workers who are trusted members of and/or have a close understanding of the community served.^25,26^ In asthma education sessions, CHWs deliver education about reducing environmental asthma triggers, including air pollution, and providing materials such as dust mite covers and high-efficiency particulate air (HEPA) filter vacuum cleaners and referring clients to resources such as smoking cessation and weatherization programs, as appropriate.^27^ They also support communication with their clients’ schools, landlords, and healthcare providers. CHW-delivered asthma programs have been demonstrated to be effective at decreasing pediatric asthma symptoms, asthma-related emergency department visits and hospitalizations, limitations on daytime activities, and missed school days.^25,28–32^

Another intervention for improving the health of children with asthma is the use of HEPA portable air cleaners.^33–44^ As reviewed recently, air cleaners with filters can substantially reduce indoor PM_2.5_ and other particulates in real-world conditions.^45,46^ Some research suggests that use of indoor air cleaners may also improve respiratory health for adults and children, but there are some inconsistencies between studies.^47^ Indoor air cleaners have important limitations. The cost of an air cleaner, the replacement HEPA filters, and electricity for continuous operation can be a barrier for some families.^47,48^ Short- and long-term adherence to air cleaner use is mixed across studies.^39,49,50^ Also, the efficacy of air cleaners varies by pollutant, housing characteristics, study setting, and adherence to air cleaner use. More research on air cleaner effectiveness in diverse settings is needed.^47^ To the best of our knowledge, there has been no study of the effectiveness of air cleaners with HEPA filters to address poor indoor air quality and pediatric asthma health in neighborhoods impacted by airport-related air pollution.

### OBJECTIVES

In the Airport, Air quality and Asthma (AAA) research study, we are conducting a randomized controlled trial to evaluate whether the use of air cleaners with HEPA filters can effectively reduce indoor air pollution and improve airway health for children with asthma living near a major airport. We hypothesize that the intervention will be associated with lower concentrations of indoor PM_2.5_, improved asthma control, and lower incidence of airway symptoms. This study is a collaboration with a county health department, leveraging an established CHW-delivered asthma education program for support on participant recruitment, community engagement, and data collection. By conducting the trial before initiation of the asthma education program, we aim to distinguish any beneficial impacts of HEPA air cleaners from those of the asthma education intervention, a challenge in prior similar studies.^44^ The AAA research study will also serve as an example of a community-engaged research project centered on partnership between academia and a local health department, with CHWs serving as important community liaisons.

## METHODS

### Trial design and study overview

The design is a parallel group, two-arm randomized superiority trial. In brief, participating households are block randomized to either an intervention group or a lower quality filter group in a 1:1 ratio overall and further balanced 1:1 within groups by household smoking status, defined as reported presence versus absence of smoking household members. At the start of the trial, an indoor air cleaner is set up in the bedroom of the participating child. The two arms of the study are distinguished by the type of filters included in the air cleaner. Air cleaners in intervention group homes are outfitted with a set of three filters (pre-filter, activated carbon filter, and HEPA filter) while those in the less effective filter group receive an air cleaner with only a pre-filter. This comparator is appropriate because very coarse particles, including pet dander and dust, will be filtered, but fine particulates, such PM_2.5_, will not; this allows us to test the potential effect of HEPA filtration specifically. Using a less effective filtration blinds participants to intervention status, mitigating possible bias in reported outcomes. All households are followed for a three-month observation period that coincides with administration of the intervention.

The AAA research study was retrospectively registered at clinicaltrials.gov (identifier: NCT07047430; registration approved July 1, 2025).

### Study setting

**Figure 1:**
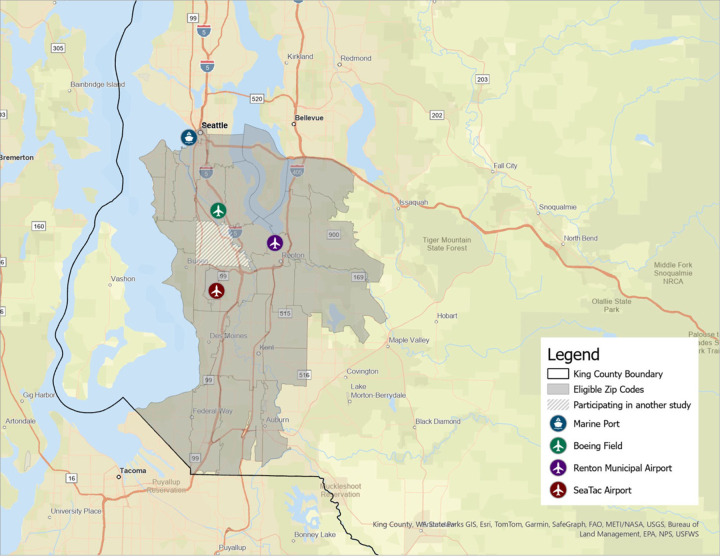
The AAA Study region. Eligibility criteria included residence in a zip code within 10 miles of the Seattle-Tacoma (SeaTac) International Airport, excluding one zip code that was excluded because it was the recruitment region for a separate study.

The study is set in South King County, Washington, in the communities surrounding the Seattle-Tacoma International Airport (Fig. 1). This region has a lower median income and a higher percentage of people of color than the rest of the county. Roughly three-fourths (74.8%) of the county’s Native Hawaiian/Pacific Islander residents and the majority of its Black/African American (72.7%) and Hispanic/Latino (57.6%) residents live in this area.^51^has a relatively high exposure to road and air traffic pollutants, including PM_2.5_ and UFP, due to emissions associated with the Seattle-Tacoma International Airport, Boeing Field (a smaller regional airport), and a high density of busy roadways and highways. In 2022, the asthma hospitalization rate for children in this region was 57% higher than for the rest of the county (59.9 vs. 38.0 per 100,000); for adults, the rate was 80% higher (26.2 vs. 14.6 per 100,000).^52,53^

### Collaborations with local health department and community-based organizations

The AAA research study is a collaboration between the University of Washington and the Public Health-Seattle & King County Asthma Program, a CHW-administered asthma intervention program based on the “Healthy Homes” model that has supported King County families for over 20 years.^30^ In 2024, the asthma program was awarded funds from the Washington state legislature for the Airports, Air quality, and Asthma (AAA) Initiative. This initiative funded both the AAA research study and a significant expansion of the King County CHW Asthma Program to serve high-need communities near Seattle-Tacoma International Airport.

To increase the program’s capacity and better serve these communities, the AAA Initiative recruited and funded four community-based organizations (CBOs) to train and deploy CHWs. The four CBOs, African Community Housing & Development, Cultivate South Park, Lutheran Community Services Northwest, and Villa Comunitaria, each had well-established CHW programs and deep connections with the diverse populations within the focus geography. The African Community Housing & Development empowers the community of African Diaspora immigrants, refugees, and their descendants by building culturally rooted health and housing stability through economic development, legal support, resource navigation, holistic education, and access to cultural arts and traditions. Cultivate South Park is a resident-led, asset-based community development organization identifying, connecting, and celebrating neighborhood gifts in the South Park neighborhood. Lutheran Community Services Northwest enhances health outcomes through partnerships, specialty medical and mental health access, health education, and culturally sensitive case management for their largely immigrant and refugee clients. Villa Comunitaria serves predominately Spanish-speaking families in economic development, citizenship and voter education, healthy communities, skills and leadership, and systems navigation. The CHWs in these organizations live within the target communities and share the population’s language and racial/ethnic background. They were trained and mentored on how to provide direct services by the experienced CHWs in the King County CHW Asthma Program.

The King County CHW Asthma Program and CBOs support the AAA research study in several ways. The CHWs facilitate recruitment and enrollment into the study, communicate with research participants, attend home visits at the beginning and end of the study, and provide culturally knowledgeable facilitation between participants and the research staff. This integration of the King County CHW Asthma Program and the research study is an important strength of the AAA approach because recruitment into the Asthma Program and the research study are conducted in parallel. The relationship between the CHWs and participating families is reinforced through the dual roles of the CHW, in participating on the research team and in administering the King County CHW Asthma Program intervention.

The CHWs also received additional education and training in environmental health from the AAA research team, including interactive training sessions on environmental justice and air pollution, with a specific focus on issues affecting the study region. CHWs were also trained on research procedures, including how to protect personally identifiable information and use of REDCap for data collection.^54,55^ The AAA research team collaborated with the CHWs to design a communication strategy, including low-burden and integrative methods for scheduling home visits. The CHWs also provided feedback on study materials, including surveys and other documents, providing a valuable perspective from the communities they serve.

### Participant eligibility criteria

**Figure 2:**
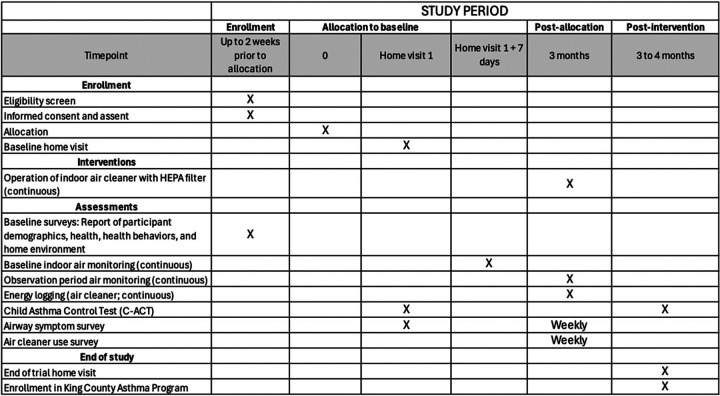
SPIRIT Diagram.

Children with asthma and one caregiver in the same household were recruited for participation in the AAA research study. Figure 2 displays the timeline of all activities, starting from screening. Inclusion criteria were: child is 6–12 years old with health care provider-diagnosed asthma; caregiver is comfortable participating in all study activities in English, including communicating with study staff by phone and completing online surveys, and is familiar with the child’s daily asthma health; residence within 10 miles of Seattle-Tacoma International Airport as determined by zip code; child resides in caregiver’s home at least five nights a week on a regular basis; and no plans to move in the next three months. Participants were excluded if the child was determined to have severe asthma at baseline, because participation in the research study requires up to a 4-month delay in initiation of the King County CHW Asthma Program. Severe asthma was defined in consultation with pediatric pulmonologists to be caregiver-reported asthma symptoms every day over the past 14 days and/or two or more inpatient hospitalizations for asthma in the past 12 months. Households were excluded if there were multiple individuals with asthma eligible for the King County CHW Asthma Program if any of the household members had severe asthma.

### Recruitment, participant consent/assent, and ethics review

**Figure 3:**
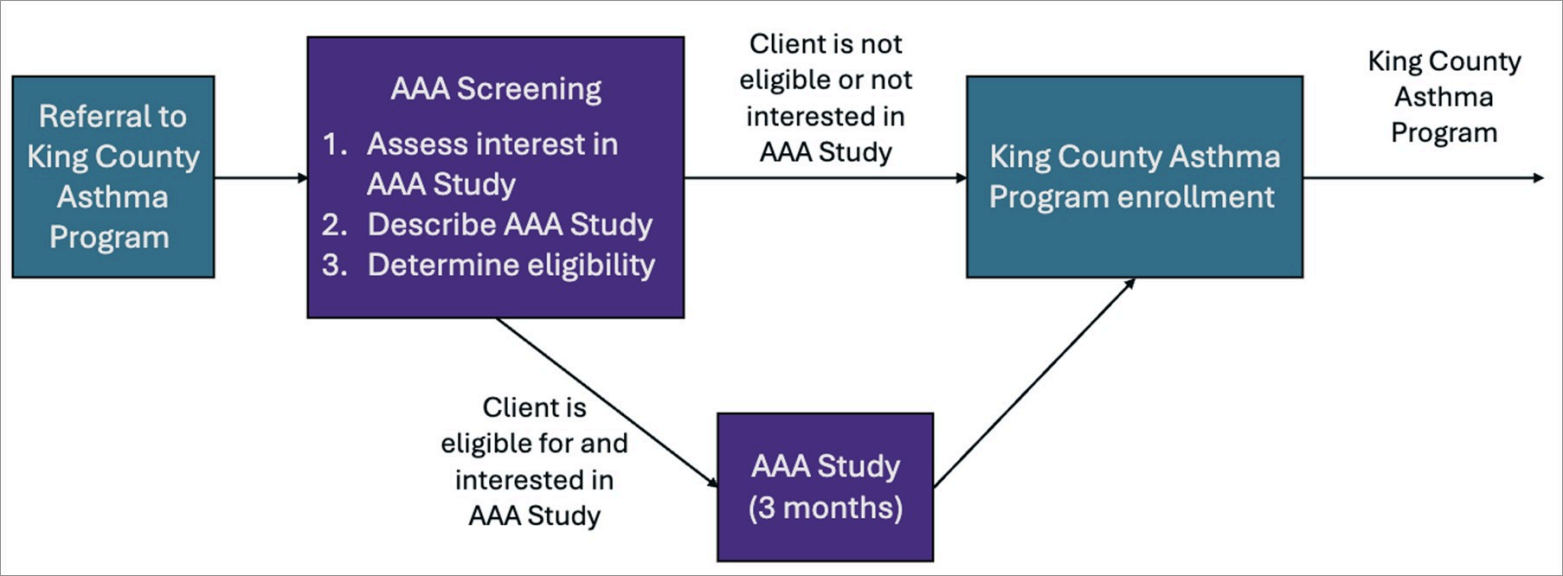
Enrollment into the King County CHW Asthma Program and the AAA Research Study. Recruitment for the AAA Research Study is nested within protocols for referral and enrollment into the King County Asthma Program. Referrals to the Asthma Program are screened for eligibility in the research study and – if eligible – given the opportunity to enroll. Those who do not enroll in the research study proceed to enroll in the Asthma Program. Those who participate in the research study are offered enrollment in the Asthma Program after completion of the study.

Enrollment into the AAA research study began June 1, 2024, and was ongoing at the time of this report. The target enrollment is N = 60 child-caregiver pairs. Recruitment is implemented as part of the referral protocol for the King County CHW Asthma Program (Fig. 3). Potential new Asthma Program clients are self-referred, referred by a health care provider, or referred by a caretaker. Each new referral is assigned to a CHW, who calls the client or a caregiver within three business days. If the individual with asthma being referred is a child 6–12 years old and lives within the focus geography, the CHW asks the caregiver permission to screen for eligibility for the AAA research study. Those who are not interested in the research opportunity or who are determined to be ineligible proceed directly to enrollment in the King County CHW Asthma Program, conducted by an AAA study coordinator. Caregivers who expressed interest in enrolling are asked by the coordinator to review and sign an online consent form and to support the enrolled child in an assent process, which includes a video describing the study. The CHW then schedules a baseline home visit for the AAA research study.

All research protocols, including the consent and assent procedures and materials, were reviewed and approved by the UW Institutional Review Board and the Research Administration and Review Committee of Public Health-Seattle King County. Confidentiality of identifiers is ensured by use of a HIPAA compliant data collection platform (REDCap). Participants are offered monetary incentives for participation, distributed after the first home visit and after the second home visit, and they are allowed to keep the air cleaner and a HEPA filter replacement kit at the end of the research study.

### Participant safety monitoring

Participant safety monitoring includes weekly review of caregiver reports of child airway symptoms (see below). Children who have 2 or more weeks of daily symptoms may experience uncontrolled, severe asthma. When this occurs, the caregiver is contacted to confirm the responses and discuss the child’s current asthma health. Researchers then present the individual case to the pediatrician consulting with the asthma education program to determine whether the child can continue participating in the research protocol.

### Trial timeline

**Figure 4:**
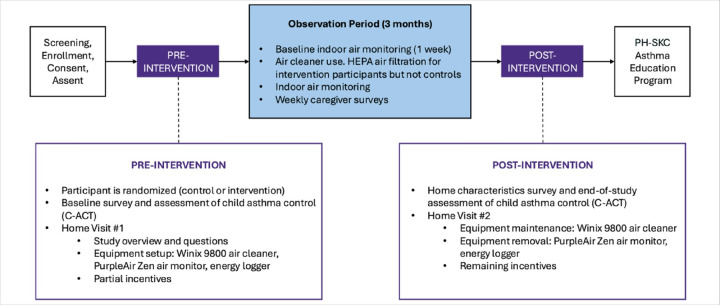
AAA Research Study Participant Timeline. There are three phases of data collection: pre-intervention, observation period (coinciding with intervention delivery to participants in the intervention arm), and post-intervention. Abbreviations: HEPA = high efficiency particulate air filtration; C-ACT = Child - Asthma Control Test; PH-SKC = Public Health-Seattle King County.

Figure 4 shows the timeline of research activities for each participant. Following recruitment into the AAA research study and allocation to treatment groups, pre-intervention (i.e., baseline) data collection of effectiveness outcomes is conducted through surveys and indoor air monitoring. A three-month observation period begins after initiation of the intervention and includes longitudinal data collection (continued indoor air monitoring and caregiver surveys on child asthma symptoms). At the end of the observation period, the baseline assessment of child asthma control is repeated. All families are offered the opportunity to enroll in the King County CHW Asthma Program after the AAA research study ends.

### Allocation procedures

After enrollment, each child is randomized to the intervention or less effective filter group using the REDCap Randomization Module by a study coordinator. Participants are block-randomized based on household smoking status, to ensure an approximately equal ratio of smoking and nonsmoking households in the two groups. Smoking is a very strong determinant of indoor air quality as well as child airway health, and balancing groups by smoking can reduce the influence of residual confounding in the case that randomization does not achieve balance between the groups. We chose not to exclude households with smokers because that would reduce the generalizability of study findings. When two children in one household were enrolled in the research study, both were randomized to the same group. Participants and CHWs are blinded to intervention status. The research team, including those who attend the home visit or manage and analyze data, are not blinded. Protocols for unblinding in the course of the trial were deemed unnecessary and not established.

### Baseline data collection (pre-intervention)

Pre-intervention data collection included surveys and indoor air monitoring, to describe the study population and estimate baseline levels of intervention outcomes.

#### Baseline surveys

Caregivers complete an online baseline survey after enrollment. Survey topics include family sociodemographics, health behaviors, child airway health history, access to health care, asthma medication use, and features of the indoor environment. Baseline surveys include the Child Asthma Control Test (C-ACT), which the caregiver completes in collaboration with the child.^56–58^ The C-ACT is a short seven-question survey used to characterize child asthma control based on parent and child report of symptoms over the prior 30 days. The C-ACT has been validated for ages 4–11 years old and shown to have good psychometric properties.^56–58^ A C-ACT score is calculated based on responses, ranging from 0 to 27, with higher values reflecting better asthma control, and a score of 19 or lower considered to reflect inadequate, or “poor,” control.^57^ Study team staff review responses on the baseline survey to address missing data or participant confusion during the study visits.

## Baseline home visit

AAA research study team members and the CHW assigned to the family conduct a home visit after enrollment. Attendance of the caregiver but not the child is required. At the start of the visit, the CHW introduces the research study team staff to the participants and requests permission to enter the home, including the child’s bedroom. The visit protocol includes an overview of study activities and discussion of participant’s questions or concerns, if any; set-up of the air cleaner, air monitor, and energy data logger in the child’s bedroom (details below); completion of the baseline survey and/or review of any questions that require additional clarification, if necessary; and demonstration of the weekly asthma symptom surveys, with completion of the first weekly survey. A brief inspection of the child’s bedroom includes measurement of its dimensions, description of carpet and fabric-covered items in the bedroom, and documentation of any existing air cleaner in the room.

While the AAA research study team is setting up equipment, the CHW engages with the caregiver (and child, if present). Topics may include the family’s experience with asthma and asthma symptoms, future opportunities for individualized asthma education and management, and relevant services and programs offered by the CHW’s community-based organization (e.g., housing services and food security programs).

### Intervention: Air cleaner installation and operation

During the baseline home visit, a Winix 9800 air cleaner is installed in the child’s bedroom. The Winix 9800 is a true HEPA air cleaner that has been Association of Home Appliance Manufacturers Verifide^®^ for rooms that are 500 square feet in size. The air cleaner has a washable fine mesh pre-filter that captures large airborne particles, an activated carbon filter that reduces volatile organic compounds and odors, and a true HEPA filter that captures 99.99% of airborne allergens as small as 0.003 microns in size. Households randomized to the intervention group (arm 1) receive an air cleaner with a pre-filter, carbon filter, and HEPA air filter pre-installed while those in the less effective filter group (arm 2) receive an air cleaner with only the pre-filter. Each air cleaner is connected to an Onset^®^ HOBO^®^ UX120–018 plug load data logger which measures and records the power and energy consumption of the air cleaner (energy logger), which will be employed to assess use of the air filter.

With input from the caregiver, the field team places the air cleaner in the child’s bedroom. Air cleaner placement is mainly based on the location and availability of a power outlet. If possible, the air cleaner and PurpleAir air monitor (described below) are placed on opposite sides of the bedroom to ensure sampling from well-mixed room air. The caregiver is instructed in the handling and operation of the air cleaner, encouraged to keep the fan on during the full 3-month study period, and told that typical fan speeds at night are *low* or *medium* fan speed. When the air cleaner is first turned on, the default setting is automatic mode, in which the air cleaner will automatically increase fan speed based on an internal particle counter. The caregiver is also instructed not to move or open the air cleaner, not to disconnect the data logger, and that there is no need to change any of the filters during the study period.

The caregiver is asked to keep the air cleaner off for a week after the baseline home visit for the collection of pre-intervention air pollution concentrations. A sticker is placed on the air cleaner as a reminder of the date, and the CHW also contacts the caregiver on that date to confirm the air cleaner is turned on.

We employ several strategies to improve adherence to the intervention protocols. Protocols are discussed with the study team, including the trusted CHW, in person at the baseline home visit, with demonstration of the equipment and with opportunities for the participant to ask questions. As described below, caregivers complete weekly surveys, which include questions about air cleaner use. In this way, they are reminded of the protocol and prompted to continue using the cleaner and, they also have a convenient opportunity to report problems with the devices if they have any. Adherence is monitored using the energy loggers as well as through weekly surveys, on which caregivers report frequency of use over the prior seven days.

Participants were not asked to restrict engagement in any other interventions or health care for their child during the trial.

### Longitudinal data collection (observation period): Indoor air monitoring and weekly symptom surveys

A three-month observation period begins the day that the caregiver confirms initiation of air cleaner operation. During this period, the caregivers are encouraged to use the air cleaner continuously. Longitudinal measures of indoor air quality and child airway health are collected throughout this period.

Indoor air monitoring of PM_2.5_ is conducted with a PurpleAir Zen air quality monitor, installed in the child’s bedroom during the first home visit, affixed to the wall or atop a shelf, depending on the availability of space. The monitor collects PM_2.5_, ambient temperature and relative humidity at 5-minute intervals, stored on the device (via microSD card) and downloaded after the monitor is retrieved at the end-of-study home visit. Data will be uploaded to study drives, grouped by daily measurement clusters. Completeness of the raw data will be assessed (e.g., percentage of expected data points), and quality assurance protocols will be applied. These protocols include removal of values with low within-room correlation (r < 0.7), exclusion of days with zero variation in measurements, and attenuation of extreme values through Winsorizing within each participant monitoring period.

Longitudinal child airway health is characterized through brief online surveys sent to caregivers on a weekly basis. Caregivers report the number of days in the prior week on which the child was coughing, wheezing, had shortness of breath, was awakened at night due to asthma and used asthma quick relief medication (e.g., albuterol inhaler).^59^ They are asked whether the child had an unscheduled clinic visit, an emergency room visit, or hospitalization due to asthma in the past seven days. Caregivers are also asked to report the number of nights of the past week the child spent at home and whether the child had a respiratory infection. Weekly surveys ask caregivers about whether the family experienced any issues with the indoor air cleaner and how frequently they used it in the past week.

### Retention and protocol for attrition or intervention deviation

We used several methods to maximize retention and data completion, including integration of the Asthma Program CHW in the baseline home visit and participant communications, weekly surveys for data collection as well as outreach, and timely follow up in the case of missing data. In the case of participant drop-out or deviation from the intervention protocol (i.e., limited use of air cleaner), we will retain all outcome data and conduct sensitivity analyses with exclusion of participants or specific periods, as appropriate.

### End of study data collection (post-intervention): Second home visit

After the observation period ends, a second home visit is scheduled. At this visit, the research team collects the air quality monitor and the energy logger. The HEPA air cleaner is also inspected and the pre-filter is cleaned by a handheld HEPA vacuum cleaner. A new black carbon is installed in air cleaners of intervention families; HEPA and carbon filters are installed in air cleaners of families in the less effective arm. All families are also provided with a new replacement filter kit and extra activated black carbon filters. The research team provides instructions for future air cleaner use and maintenance and asks about the family’s overall experience in the study and ease of use of the air cleaner.

### Primary and secondary intervention outcomes

The effectiveness of the intervention to reduce indoor air pollution and improve the health of children with asthma is assessed through several primary and secondary outcomes. Primary outcomes related to air quality include the average indoor PM_2.5_ in the child’s bedroom as well as the ratio of indoor to outdoor PM_2.5_, both averaged across the three-month intervention period. Outdoor PM_2.5_ will be estimated from regional regulatory air monitors and PurpleAir sensor data, as available. Primary health outcomes include the change in child ACT score between baseline and post-intervention as well as the total count of symptom-days in the observation period, with “symptom-day” defined as a day on which any asthma symptom or medication use was reported. Several secondary health outcomes will be analyzed in exploratory analyses, including: count of unscheduled clinic visits, ER visits and hospitalizations for asthma during the observation period; count of symptom-days, broken down by different type of symptom (coughing, wheezing, shortness of breath) and count of asthma medication use days; and count of respiratory infections in the observation period.

### Data analysis plan and power calculations

Descriptive analyses will be conducted to describe study population characteristics, concentration of indoor air pollutants (baseline and observation period), and child asthma health outcomes. Distributions of each variable will be summarized overall and stratified by intervention, with continuous variables presented as means, medians, standard deviations and interquartile ranges and categorical variable summarized as counts and percentages within each category. In order to evaluate completeness of randomization between the intervention and less effective groups, t-test and chi-squared tests will be conducted to determine whether there is a significant difference in characteristics between groups.

All analyses of intervention effectiveness will be conducted with an intent-to-treat approach. The effect of the intervention on indoor air quality will be estimated through multivariate regression with robust standard errors, with average calibrated pollutant concentration as the dependent variable and group status (intervention or less effective arm) as the independent variable. Regression models will include adjustment for baseline pollutant concentration, measured in the seven days prior to intervention initiation, outdoor PM_2.5_ concentrations, season and any predictors of indoor air quality that are not balanced between the intervention and less effective groups. Sensitivity analyses will include repeating all analyses with exclusion of participants with relatively low compliance rates of indoor air cleaner usage, determined by self-report (weekly surveys) and energy monitor data.

The effect on count of symptom-days across the intervention period will be assessed using Poisson regression with robust standard errors, with count of symptom-days during the intervention period as the dependent variable and group status as the dependent variable.^59,60^ The regression model will be adjusted for season and for participant or home characteristics that are risk factors for asthma symptoms or exacerbations and not balanced between groups. Secondary health outcomes (count of unscheduled clinic visits, ER visits and hospitalizations for asthma during the observation period; count of symptom-days, broken down by different type of symptom (coughing, wheezing, shortness of breath) and count of asthma medication use days, will be analyzed using the same approach, in separate models. The time at risk of event (i.e., symptom-days) will be estimated for each participant using the number of completed weekly surveys. In sensitivity analyses, weeks in which the child spent three or more days away from home will be excluded.

The target enrollment is N = 60 children (N = 30 per arm). For all power calculation, we assumed that significant confounders of this relationship are balanced between groups by randomization or addressed by adjustment. We estimated the power to detect an effect on the primary outcome related to indoor air quality, reduction in indoor PM2.5, using a simple two-sample t-test comparing the less effective and intervention groups on the within-subject differences between baseline and post-intervention indoor PM_2.5_. Based on prior literature,^38,39,50,61,62^ we estimate that the intervention effect on PM_2.5_ will range from 35–60% with a coefficient of variation (CV) from 60–80%. Power was calculated for these ranges of effect size and CV in a sample size of 30 homes per arm, demonstrating sufficient power (greater than 0.80) for all scenarios. We similarly estimated the power to detect an effect on two primary outcomes related to child airway health. We estimated that a sample size of N = 60 would have power of 0.81 to detect a 2-point difference in the C-ACT associated with the intervention. We also estimated power to detect a reduction in the count of symptom-days. Assuming a Poisson distribution, a percentage of symptom days in the less effective group of 15%, a sample size of N = 60 provides power of at least 0.80 to detect an effect size of 18% or higher.

### Audits and approach to protocol amendments

Trial conduct is reviewed and discussed by staff on a biweekly basis, independent of the sponsor. Important changes to the protocol will be discussed at these team meetings and submitted to IRBs for review as necessary. Changes that impact participants will be communicated to them *via* assigned CHW. Updates to the trial registry will be made as needed.

### Dissemination of results and data sharing

Trial findings will be communicated to the sponsor in a final report. We will also return results to the community organizations in meetings and/or in written reports, based on their preferences. Findings will also be presented in publications submitted to peer-reviewed journals.

Deidentified data are available upon request, pending completion and approval of a data sharing agreement with Public Health Seattle King County. The full protocol and all statistical code will also be available upon request.

## DISCUSSION

To the best of our knowledge, the AAA research study is the first intervention trial evaluating the effectiveness of indoor HEPA air cleaners to improve indoor air quality and airway health for children with asthma residing in air traffic-impacted communities. The study is set in a region with relatively high exposures to traffic- and airport-related air pollution, pediatric asthma prevalence and hospitalization rates for asthma-related conditions, and socioeconomic disadvantage. Several strengths of the study increase the chances that findings can be translated to effective, feasible and sustainable asthma interventions in the future, with the ultimate objective of reducing child health inequities that result from disproportionate exposures to outdoor and indoor air pollution.^63–65^

A notable aspect of the study design is close collaboration with the local health department and a CHW-delivered asthma program. The proposal for this research study was developed in partnership with leadership of the King County CHW Asthma Program, and all study procedures were drafted and evaluated with feedback from the CHWs who are serving the target community. Administrative staff are the first point of contact for potential research participants as they are referred to the King County Asthma Program, introducing the research opportunity to caregivers of children who are age-eligible and conducting an eligibility assessment. A CHW is then assigned to the participant and becomes their main point of contact. Recruitment has been successful to date, likely reflecting the close relationships that CHWs and participating community-based organizations can form with community members.^66–68^ CHWs have also been integral in AAA community outreach activities, raising awareness of both the King County CHW Asthma Program and the AAA research study through a variety of mechanisms, including in-person events and social media engagement. The CHW attends AAA study home visits along with research study staff, and the same CHW initiates the King County CHW Asthma Program seamlessly after the completion of the research study whenever possible. When the entire study is finished, CHWs will participate in efforts to disseminate findings to the community. Other community-engaged research studies have successfully leveraged relationships between CHWs and other trusted advocates and the study community in similar ways.^44,67,69^

There are several methodological strengths of the AAA study. A randomized controlled trial design, relatively rare in the field of environmental health, minimizes the influence of potential confounders on estimates of intervention effect. We blinded participants to intervention status by installing air cleaners with “less effective” filtration for participants not randomized to the intervention, which reduces the potential of bias in reported outcomes. Most other intervention studies of indoor air filtration in schools or homes of children with asthma did not use this approach.^38–44^ Another strength is use of energy monitors to collect objective data on use of portable air cleaners, which allows descriptive characterizations of patterns in adherence to air cleaner use, an important factor in the effectiveness of air cleaners in reducing pediatric asthma morbidity, as well as sensitivity “per protocol” analyses, accounting for the frequency of air cleaner use. Few studies of portable air cleaners and respiratory health have utilized energy monitors, a notable limitation.^44^

While the AAA study is conducted in series with a CHW-delivered asthma program, the trial was designed to evaluate the effect of portable air cleaners independent of the expected beneficial effects of the asthma program. Therefore, we conducted the trial prior to initiation of the asthma program rather than concurrently, in contrast to other studies which were challenged to distinguish beneficial effects of the intervention from that of the asthma education program.^44,70^ The AAA study assesses multiple asthma health outcomes, including longitudinal reports of asthma symptoms and medication usage on a relatively short time scale (weekly), providing a detailed characterization of time-varying asthma health. This is important because the respiratory health of children with asthma can vary substantially from week to week, and factors such as viral infections, stress, or changes in activity can greatly influence asthma health and reduce statistical power in studies relying on a small number of respiratory health assessments (e.g., baseline and post-intervention). Repeated measurement of asthma health will provide characterization of within- and between-participant variability in health and, further, support analyses of time-varying air pollution exposure, as measured by indoor air monitors, and short-term associations with asthma symptoms and medication use.

The AAA research study also faces challenges and limitations. Trial recruitment is conducted in the context of referrals into the King County CHW Asthma Program. The capacity of this program has been limited in recent years due to a shift from providing services to training and dissemination as well as impacts of the COVID pandemic. After receiving funding to expand services and the support of the contracted community-based organizations, the program had to re-initiate referral networks and outreach efforts. These factors limited the pace of trial recruitment. Despite this challenge, the integration of trial protocols into the Asthma Program is a major strength, as described above. Another potential limitation is the possibility of missing data in repeated weekly surveys for caregivers during the three-month intervention period. Surveys were designed to be low burden for participants (short and easily accessible), and staff demonstrated the surveys to caregivers at the time of the first home visit, to increase participant familiarity with longitudinal data collection. Other limitations include the exclusion of children with severe asthma, which limits generalizability of study findings; however, exclusion was necessary in order to avoid delaying health care for children most at risk of serious exacerbations.

## Supplementary Files

This is a list of supplementary files associated with this preprint. Click to download.

- SPIRITChecklist.docx

## Data Availability

De-identified information on indoor and outdoor air quality over the course of the study period will be shared through an open-source publication. De-identified weekly symptom questionnaire will be shared, however identifiable participant information including age, gender/sex and home address will be redacted. A data management plan is available through dmptool.org, allowing researchers to identify and request data elements that are not available through open-source mechanisms. Access to the full identified dataset will be maintained by Dr. Austin, and requests to provide de-identified components beyond what is publicly available will be through direct request.

## Abbreviations

AAA: Airport, Air quality, and Asthma
CHW: community health worker
CBO: community-based organization
C-ACT: child asthma control test
ER: emergency room
HEPA: high efficiency particulate air
PM: particulate matter
PM_2.5_: particulate matter of 2.5 microns or less in aerodynamic diameter
REDCap: Research Electronic Data Capture
TRAP: traffic-related air pollution
UFP: ultrafine particulate matter
UW: University of Washington
